# Supplementary material for: Ivermectin inhibits extracellular vesicle secretion from parasitic nematodes
Source: J Extracell Vesicles. 2020 Dec 10;10(2):e12036. doi: 10.1002/jev2.12036 (PMC7726798; doi:10.1002/jev2.12036)
Supplement: Supplementary file 1 — Supporting information. [file JEV2-10-e12036-s001.docx]

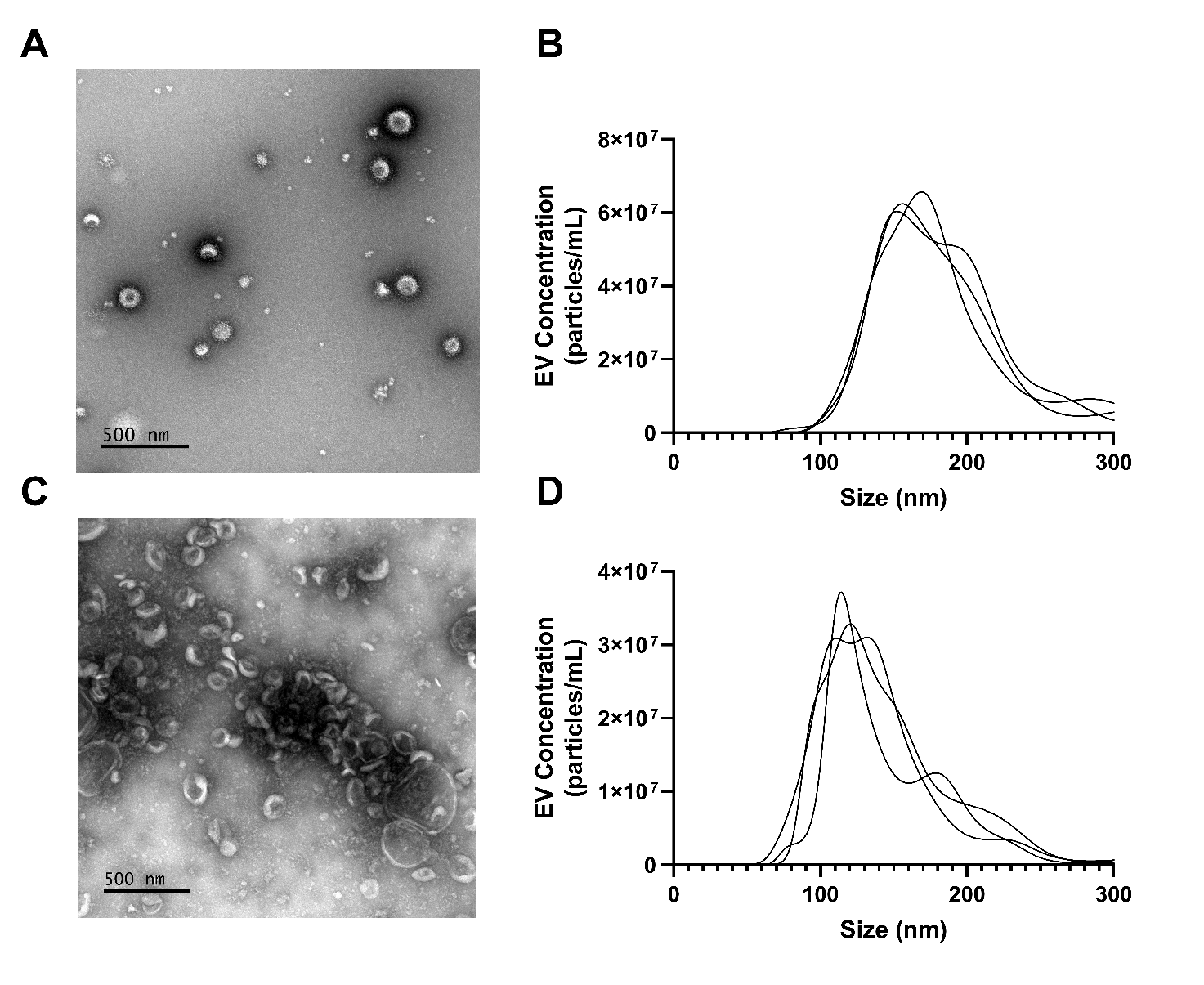


**Supplemental Figure 1.** **Validation of EVs via transmission electron microscopy (TEM) and NTA**

Adult Female *A. suum* or *B. malayi* mf were cultured and spent media was collected every 4 hrs for up to 72 hrs. EVs were isolated via differential ultracentrifugation as described. A two µl aliquot of purified EV preparation was placed onto a carbon film grid for 1 min. The drop was wicked to a thin film and two µl of uranyl acetate (2% w/v final concentration) was immediately applied for 30 sec., wicked, and allowed to dry. Images were taken using a 200kV JEOL 2100 scanning and transmission electron microscope (Japan Electron Optics Laboratories, LLC, Peabody, MA) with a Gatan OneView camera (Gatan, Inc. Pleasanton, CA). In addition, EVs were quantified using NTA. Representative electron micrographs of *A. suum* (A) and *B. malayi* mf (C) EVs. Representative NTA quantification trace of *A. suum* (B) and *B. malayi* mf (D) EVs.


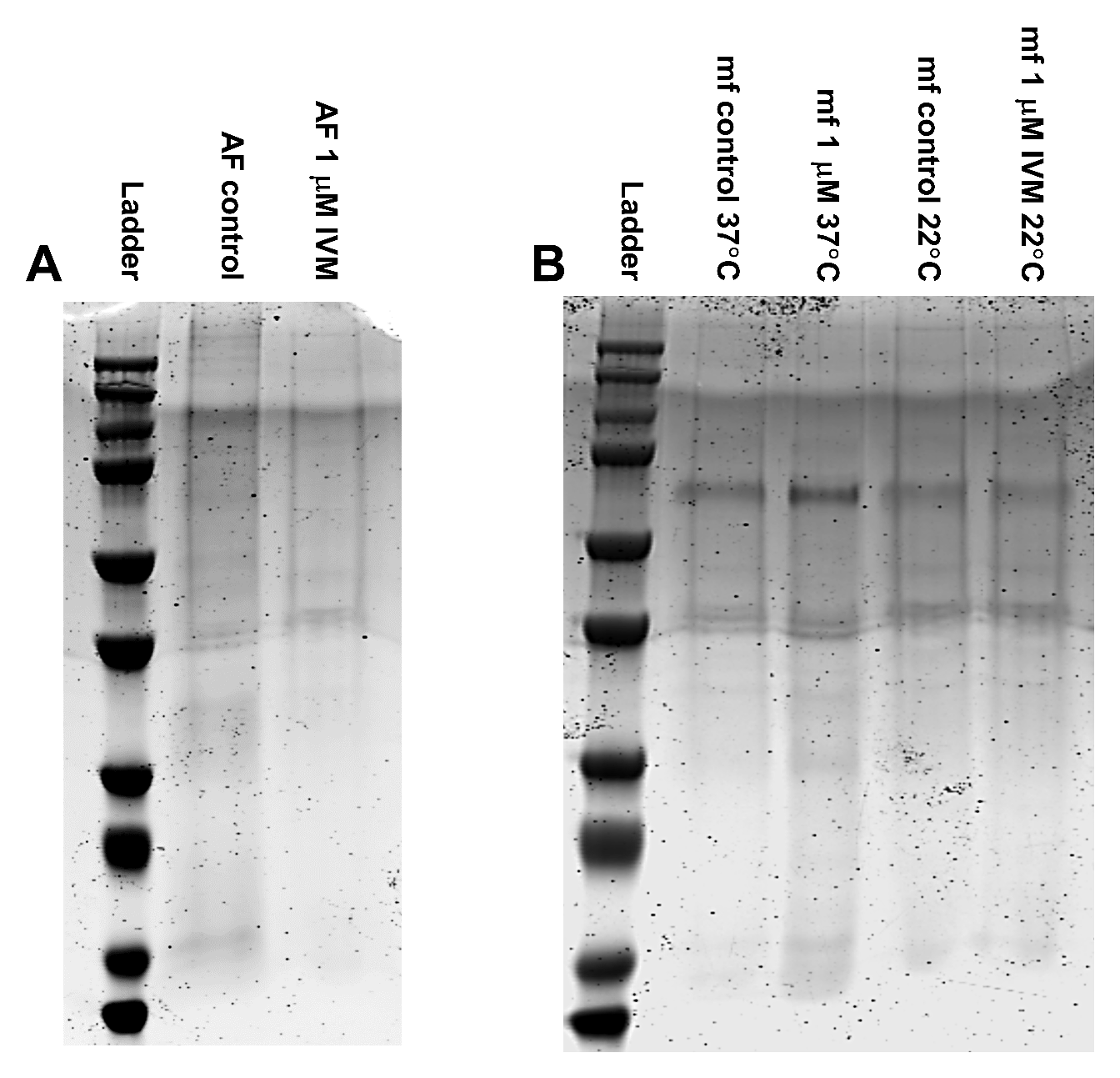


**Supplemental Figure 2.** IVM effect on ES protein secretion

15 *Brugia malayi* adult female (AF) were cultured with or without 1 µM ivermectin at 37°C for 24 hours. In addition, 1 million microfilariae (mf) were cultured either at 37°C or 22°C with or without 1 µM ivermectin for 24 hours. After spent media was collected and filtered through a 0.2 μM PVDF membrane filter (GE Healthcare) excretory-secretory proteins were concentrated using an Amicon Ultra 3000 Da Centrifugal Filter (SigmaAldrich) and washed with PBS. Protein concentration was determined using a Qubit Fluorometer (Thermofisher) according to manufacturer’s instructions. 7.5 µg and 1.8 µg of total ES protein was loaded for adult female and microfilariae samples, respectively. There were no clear differences in ES protein secretion between 1 µM IVM and control *B. malayi* (A) adult females and (B) microfilariae parasites as visualized by Coomassie Blue staining.
